# The pathogenesis of idiopathic normal pressure hydrocephalus based on the understanding of AQP1 and AQP4

**DOI:** 10.3389/fnmol.2022.952036

**Published:** 2022-09-20

**Authors:** Zitong Zhao, Jian He, Yibing Chen, Yuchang Wang, Chuansen Wang, Changwu Tan, Junbo Liao, Gelei Xiao

**Affiliations:** ^1^Department of Neurosurgery, Xiangya Hospital, Central South University, Changsha, China; ^2^Diagnosis and Treatment Center for Hydrocephalus, Xiangya Hospital, Central South University, Changsha, China; ^3^National Clinical Research Center for Geriatric Disorders, Xiangya Hospital, Central South University, Changsha, China; ^4^Department of Pediatrics, Xiangya Hospital, Central South University, Changsha, China

**Keywords:** iNPH, AQP1, AQP4, hydrocephalus, pathogenesis

## Abstract

Idiopathic normal pressure hydrocephalus (iNPH) is a neurological disorder without a recognized cause. Aquaporins (AQPs) are transmembrane channels that carry water through cell membranes and are critical for cerebrospinal fluid circulation and cerebral water balance. The function of AQPs in developing and maintaining hydrocephalus should be studied in greater detail as a possible diagnostic and therapeutic tool. Recent research indicates that patients with iNPH exhibited high levels of aquaporin 1 and low levels of aquaporin 4 expression, suggesting that these AQPs are essential in iNPH pathogenesis. To determine the source of iNPH and diagnose and treat it, it is necessary to examine and appreciate their function in the genesis and maintenance of hydrocephalus. The expression, function, and regulation of AQPs in iNPH are reviewed in this article, in order to provide fresh targets and suggestions for future research.

## Introduction

Normal pressure hydrocephalus (NPH) is a clinical condition characterized by gait abnormalities, cognitive impairment, and urinary incontinence. Clinically, it is often divided into two categories: secondary NPH (sNPH), often secondary to diseases with clear causes, such as craniocerebral trauma, subarachnoid hemorrhage, intracranial infection, encephalitis; and idiopathic NPH (iNPH), which has no definite etiology, mainly occurs in adults, and has a high incidence among the elderly. Misdiagnosis with Alzheimer's, Parkinson's syndrome, or a combination of these is common. The pathophysiology of iNPH is currently unclear. The basic theory of intracranial intravenous system compliance reduction is demonstrated by decreased cerebrospinal fluid (CSF) pulsation and impaired cobweb particle function, influencing CSF flow and absorption. The pathogenesis of iNPH is linked to irregular CSF circulatory dynamics based on circulation theory, inflammatory responses, osmosis abnormalities, and lymphatic drainage disorders based on penetration theory. Aquaporins (AQPs), which are responsible for CSF penetration and lymphatic outflow, are also involved in creating iNPH (Leinonen et al., 2021; Wang Y. et al., 2021).

AQPs are transmembrane channels that specifically transport water through their formation of an integral membrane protein. Totally, 13 AQP members (AQP0 to AQP12) have been found in mammals and amphibians. These proteins are tiny and intact membrane molecules with a molecular weight of about 30 kDa/monomer. There are currently 13 water-channel proteins that can be cloned, and they are found in every organ and gland in the body. The central nervous system contains five AQPs: AQP1, AQP4, AQP7, AQP9, and AQP11, with AQP1 and AQP4 having the greater concentration and AQP4 having the greatest concentration. The role of AQPs in CSF circulation, formation, and absorption of CSF, along with related mechanisms in cerebral edema and hydrocephalus, is being investigated.

Water is transported across primary ventricular fluids *via* AQPs: between CSF (containing ventricles and subarachnoid space), brain material (intracellular and extracellular), and intra-brain. CSF is a fluid found in the subarachnoid space and ventricles with a total volume of around 150 ml, of which 125 ml is in the substrate (Hodler et al., 2020). Net filtration and adsorption of water and solute in the interstitial space maintains CSF content (Papadopoulos and Verkman, 2013; Iliff et al., 2015; Nakada and Kwee, 2019). CSF enters the brain in the Virchow–Robin space of the para-arterial space and then is absorbed into the venous blood flow in the para-venous space (Iliff et al., 2013; Rasmussen et al., 2018). Whereas, water channels regulate water exchange, AQPs passively allow two-way water transport based on static water pressure and osmotic pressure (Papadopoulos and Verkman, 2013; Rasmussen et al., 2018).

AQP1 and AQP4 are the most abundant proteins in the mammalian central nervous system.

AQP1 is a 28 kD channel-forming integral protein widely distributed in the cell's plasma membrane in the form of tetramers, with each monomer (one AQP molecule) capable of generating a separate functional water channel. Because of its structural characteristics, AQP1 is highly permeable to water molecules, allowing 3,109 water molecules to flow through every second through a single AQP1 molecule. On the CSF side of the choroid plexus (CP), AQP1 is mainly expressed in epithelial cells and, together with carbonic anhydrase and the sodium pump on the basal side, plays a role in the production of CSF.

The most abundant aquaporin in the brain, spinal cord, and the optic nerves is AQP4, which regulates the brain's distribution and balance of water. It is found primarily in the astrocyte end-feet in the blood–brain barrier CSF–astrocyte boundary membranes, and ventricular membranes. As the most abundant water channel, AQP4 governs the water steady state. The AQP4 monomer is 30 kDa in molecular weight and consists of six cross-film spirals and two incomplete cross-film spirals. Each monomer contains a central water hole. AQP4 is expressed as a tetramer in the cell membrane. AQP4 is classified into two subtypes, M1 and M23, and its translation begins earlier than that of methionine M1 (323 amino acids) or methionine M23 (301 amino acids). A quadrangle composed of M23 forms a higher order structure known as an orthogonal array of particles, a crystal-like supermolecular component of a mass membrane (Wang et al., 2022). Although the biological significance of orthogonal arrays of particles has not been determined, it has been hypothesized to increase water permeability, alter cell–cell adhesion, and induce AQP4 polarization. Its highly efficient transmembrane and transcellular water transport channels contribute to the formation and removal of cerebral edema, as well as the generation and absorption of CSF. AQP1 and AQP4 are water channels located in the areas above and are associated with eliminating cerebral edema and hydrocephalus (Castañeyra-Ruiz et al., 2013).

In the current study, patients with iNPH had higher levels of AQP1 expression and lower levels of AQP4 expression, while AQP4 expression has grown with little polarization in chronic and age-related experimental models (de Laurentis et al., 2020).

AQPs may prove to be a useful diagnostic tool. The sole treatment option for hydrocephalus is treatment with a CSF shunt, but there is a risk of fatal complications (Raneri et al., 2017; Hodler et al., 2020). Thus, they are critical for diagnosing hydrocephalus. Diagnosis of iNPH remains challenging. However, detecting AQPs in CSF as biomarkers for identifying iNPH and separating these individuals from those with Alzheimer's disease is impossible, according to recent studies, at least given the detection levels afforded by commercial ELISA kits. AQP1 and AQP4 shedding into the CSF does not appear to occur in any of these illnesses, at least not at measurable quantities by ELISA (Hiraldo-González et al., 2021). A potentially useful method is to provide quantitative CSF samples during aspiration tests (de Laurentis et al., 2020). Although there are no extremely sensitive and specific CSF biomarkers that may predict the efficacy of a CSF shunt intervention, several studies have shown the potential of A42, p-tau, total tau, NfL, and LRG (Nakajima et al., 2021; Thavarajasingam et al., 2022). Additionally, AQPs may be used as therapeutic targets (Rizwan Siddiqui et al., 2018; Ding et al., 2019). Their exact activity enables alternating inhibition and activation of medicines, administered intrauterine or orally. Early intervention in the prodromal phase of iNPH was shown to aid strong cognitive and mobility function as well as social involvement capacity in the long-term experimental investigations. Its ability to maintain long-term cognitive function implies that it can help prevent dementia. Establishing an early diagnosis method for iNPH is desirable in order to provide early intervention for iNPH (Kajimoto et al., 2022).

It is critical to investigate and understand the role of aquaporin in the formation and maintenance of hydrocephalus to ascertain the causes of iNPH, leading to diagnosis and treatment. This article summarizes the current state of research on iNPH and findings on AQPs to identify future targets and research directions.

## The pathogenesis of iNPH

The exact cause of iNPH is still unknown, despite the fact that various mechanisms have been theorized to contribute to its development. Previously, it was believed that ventriculomegaly and other neurological abnormalities in iNPH were first caused by a change in the dynamics of the CSF. A proxy for CSF pulsatility may be the aqueduct stroke volume, which is measured using phase-contrast magnetic resonance imaging (PC-MRI) and is defined as the average of the flow volume *via* the aqueduct during diastole and systole. Aqueduct stroke volume levels in patients with iNPH are higher than in healthy controls (Qvarlander et al., 2017; Yin et al., 2017). Reduced arterial pulsatility and decreased intracranial compliance may be related to increased CSF pulsatility. Under normal circumstances, pressure gradients cause the CSF in the subarachnoid space to be reabsorbed through the arachnoid granulations and brought up into the superior sagittal sinus (Bothwell et al., 2019). In individuals with iNPH, the normal CSF drainage is disturbed. The resistance to CSF outflow (Rout), which has been widely utilized to diagnose iNPH and identify candidates for shunt operations, is pathologically high in these individuals (Malm et al., 2011).

The bulk flow of CSF into the brain is facilitated by the recently discovered glial–lymphatic (glymphatic) pathway along the para-arterial space, the interstitial space, and ultimately into the paravenous space. Intact AQP4 channels, proper arterial pulsation, and proper sleep are all necessary for the glymphatic system to operate normally (Rasmussen et al., 2018). Individuals with iNPH have a compromised glymphatic system. Reduced clearance of neurotoxic chemicals like beta amyloid and hyperphosphorylated tau is a direct result of the glymphatic system disruption (HP tau) (Eide and Ringstad, 2019). Accumulation of these neurotoxic chemicals may cause astrogliosis and neuroinflammation in addition to impairing the physiological functioning of neurons (Angelucci et al., 2019).

In addition, aberrant CSF dynamics cause ventricular hypertrophy, which increases mechanical stress on the parenchyma and blood vessels, leading to hypoperfusion and subsequent hypoxia. Hypoperfusion may also cause a number of pathological abnormalities to brain tissue, such as gliosis, neuroinflammation, and impairments to the blood–brain barrier. They are each acknowledged for their contributions to INPH. In the sections that follow, we'll analyze it in view of CSF overproduction and discharge disorder and investigate how AQP1 and AQP4 may be involved.

## AQP1 and hydrocephalus

The polarized expression of AQP1 in humans has also been demonstrated to play a role in CSF secretion. Existing experimental research has demonstrated a strong correlation between the development of hydrocephalus and the overexpression of AQP1. There is still a need for additional research despite evidence in iNPH patients, but numerous investigations have consistently shown that AQP1 controls water molecule distribution. The apical membrane of choroid plexus epithelial cells displays a significant amount of AQP1 expression. CSF production can be decreased by down-regulating AQP1 in the apical membrane. Therefore, specific AQP1 inhibition or down-regulation and strengthening of this beneficial negative feedback can further limit CSF secretion, reduce intracranial pressure, and postpone the pathological progression of hydrocephalus, which may also be a promising therapeutic target.

### Regulation of AQP1 on CSF circulation

AQP1 is predominantly present on the apical membrane of plexus epithelial cells (on the CSF side), with a tiny amount on the outside (near the side of the blood vessel). CSF is produced and secreted by CP epithelial cells. Some cells of the chamber membrane can also exude CSF in trace amounts, although the majority is produced by the CP epithelial cells. Regarding its transcellular transport of water molecules during CSF formation, the apical AQP1 has a relatively wide distribution. The synergy between Na, K-ATPase and AQP1 in the CP is presently the critical molecular pathway for water transfer in the ventricle and CSF creation model. Because AQP1 in the apical membrane allows water to flow in the direction of the permeation gradient, it is vital to maintain the permeability of the apical membrane. Reduced AQP1 expression on the CP's surface may account for how water molecules pass through the membrane of transmembrane CP cells and into the ventricle where they create CSF and cause hydrocephalus. According to the study, AQP1's polarized expression pattern is also involved in human CSF generation. AQP1 has been found in astrocytes surrounding cerebral arteries in various neurological disorders, including multiple sclerosis, traumatic brain injury, cerebral infarction, epilepsy, viral infections, and Alzheimer's disease. Healthy CSF contains AQP1, whereas patients with neuro-inflammatory diseases such as bacterial meningitis have elevated AQP1 levels (Blocher et al., 2011). Additionally, AQP1 is located in the blood–brain barrier, which may explain why AQPs are implicated in cerebral edema clearance.

Verkman (2008a) found that *AQP1* knockout animals had a 20% decrease in CSF fluid production and a 56% decrease in intracranial pressure compared to wild-type mice. The study discovered that when the *AQP1* gene was deleted, aquaporin expression in choroid plexus epithelial cells and CSF secretion decreased in mice, AQP expression in the nephropathic tube decreased, because AQP1 relies on decreased cross-cell reabsorption of primary urine, which results in decreased blood volume, sinus pressure, and CSF reflux. Smith et al. (2007) described an instance of communicating hydrocephalus. CP immunohistochemistry showed that the expression of AQP1 on the apical membrane of the choroid plexus epithelial cells of the ill choroid plexus was lower than that of normal children of the same age.

In conclusion, when hydrocephalus occurs, AQP1 on the to apical membrane of the choroid plexus epithelial cell enters the cytoplasm *via* endocytosis, decreasing CSF production and secretion and compensating for the decreased CSF secretion to maintain relative intracranial pressure stability, which may be a protective mechanism of the body.

However, the efficacy of this stabilizing mechanism is not without defects, as demonstrated by Jeon et al. (2017), who found (Rasmussen et al., 2018) protein in the CP of acute hydrocephalus rats using a kaolin-induced hydrocephalus paradigm. They reported abrupt reduction of AQP 1 protein expression in the CP of rats with acute hydrocephalus, increasing at later stages, following the same trend as AQP1 mRNA expression. The quick decline in AQP1 expression during the early stages of hydrocephalus is consistent with a decrease in CSF compensatory production, corroborating our theory. On the other hand, later expression levels in the experimental group were considerably higher than in the control group. The researchers believed that this was related to additional mechanisms of CSF secretion, such as neuroendocrine function (AQP1 expression may be enhanced in response to stress-induced neuroendocrine regulators) (Paul et al., 2011). Additionally, this late upregulation of expression must be confirmed. Increased expression of CP AQP1 may increase the number of water channels activated by an atrial natriuretic factor or other cGMP-producing factors in late hydrocephalus, hence decreasing CSF generation. Alternatively, cGMP-producing substances may impede the choroidal Na, K-ATPase action, resulting in decreased fluid secretion (Eide and Ringstad, 2019).

The failure of the stabilizing mechanism, which causes an increase in cerebral fluid and further aggravates stagnant fluid, may be the cause of the later increase in expression. When Wang et al. (2011) examined the brain tissue of kaolin-injected mice to create an animal model of hydrocephalus, they revealed that a substantial number of AQP1 immune granules were present in the apical membrane of CP epithelial cells in the control group. This immunological granule is normally only found in the mitochondria in the cytoplasm, whereas in the experimental group, AQP1 was abundant in both the cytoplasm and the cell membrane. Apart from mitochondria, the cytoplasm contained AQP1 immune gold granules such as vesicles and lysosomes, and almost half of AQP1 entered the cytoplasm *via* the cytomembrane. Simultaneously, when AQP1 gene knockout animals were compared to wild-type mice, it was revealed that the *AQP1* gene knockout mice's baseline ventricular area was much lower than that of wild-type mice. When lateral ventricle injection of kaolin was used to induce hydrocephalus formation, AQP1 mice in the gene deletion group exhibited a 30% reduction in ventricle dilation, a faster weight recovery following surgery, and significantly fewer side effects such as aberrant behavior.

### AQP1 in iNPH

There is a dearth of studies on AQP1 markers in patients with iNPH, and Castañeyra-Ruiz et al. (2016) found no significant increase in AQP1 levels in CSF compared to a control group. However, there were fewer cases, and in four of the six cases studied, these levels were significantly higher than those of ten control subjects, and all samples in the first quartile were controls, indicating a significant relationship between controls and low AQP1 values.

**Figure 1:**
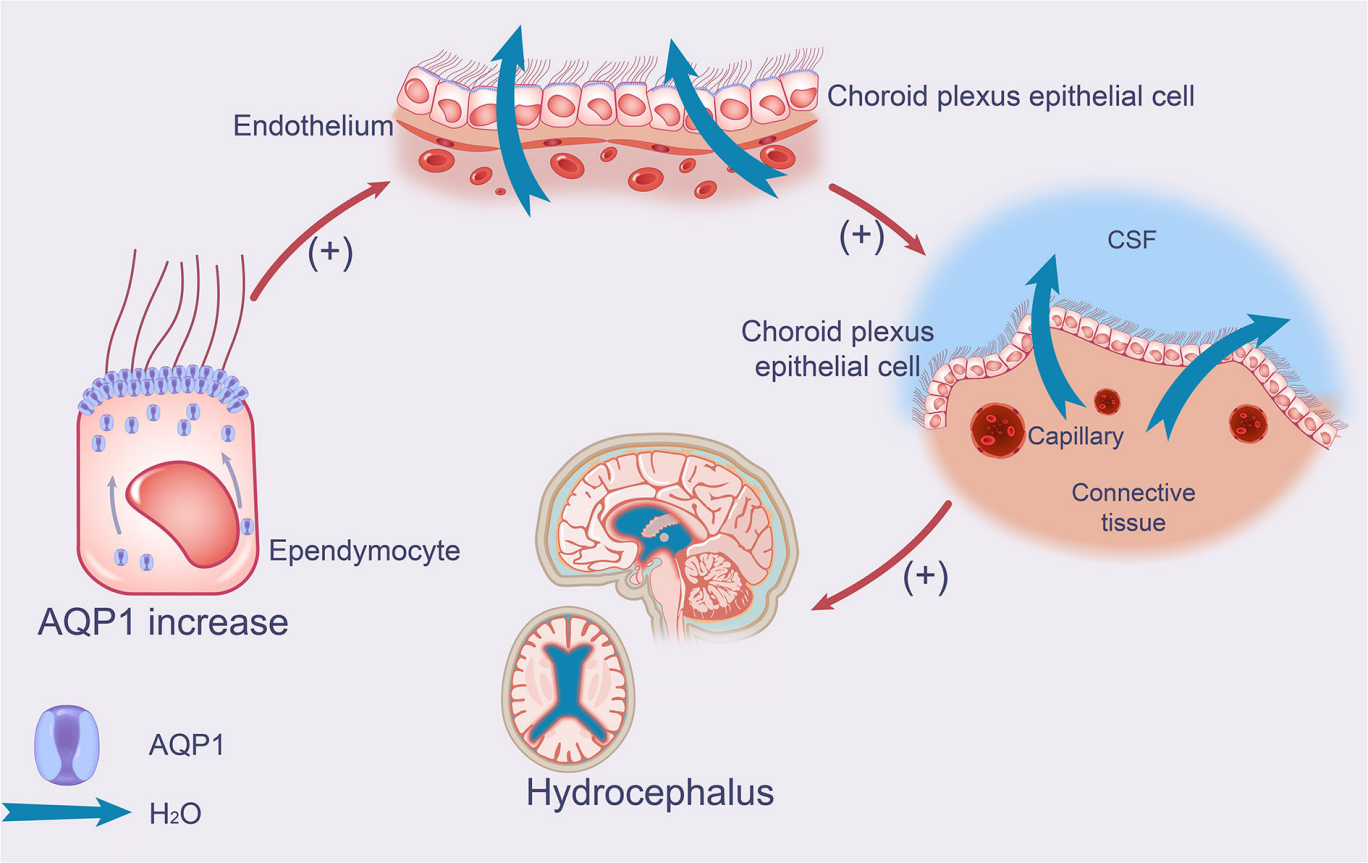
Effect of AQP1 on production of CSF in iNPH. The schematic drawing demonstrates increases in AQP1 and its function in iNPH, which leads to increased CSF production. AQP1 increases the production and secretion of CSF in the apical membrane of choroid plexus epithelial cells by accumulating in the ependymal membrane of ependymal cells. AQP1, aquaporin 1; iNPH, idiopathic normal pressure hydrocephalus; CSF, cerebrospinal fluid.

The apical membrane of choroid plexus epithelial cells expresses a high level of AQP1, and down-regulation of AQP1 in the apical membrane can result in decreased CSF production. Additionally, AQP1 can decrease blood volume and intracranial pressure by inhibiting primary urine reabsorption in the proximal convoluted tubule. These factors justify further investigation of AQP1 as a potential novel target for the treatment of hydrocephalus. Thus, selective inhibition or down-regulation of AQP1 expression, combined with enhancement of this protective negative feedback loop, can further reduce CSF secretion and intracranial pressure and delay the pathological process of hydrocephalus (Figure 1).

### The expression and regulation mechanism of AQP1

The nervous system contains a high concentration of AQP1, and various variables regulate its expression, including hormones, osmotic pressure, hypoxia, ischemia, and medications. Similar to the regulation of other proteins, cell signal transduction plays a critical role in the regulation of AQP1 expression. Numerous influencing factors can modulate AQP1 expression by activating or inhibiting the appropriate transduction pathway (Wang C. et al., 2021).

#### Activity regulation of AQP1

Phosphorylation is the most common approach for altering the function of proteins at the protein level. This also applies to AQP1, as protein phosphorylation affects AQP transport, control, and redistribution. As a result, phosphorylation is a critical strategy for modulating AQP1 function. The structural properties of AQP1 demonstrate that the nap structure, ar/R region, and sections of amino acid sequence or domain segments in the center hole all contribute to the protein's transport function; changes to these structures can directly or indirectly modify its permeability. AQP1 comprises four potential phosphorylation sites in humans and mice: S236-PKA phosphate sites, T159/T239-PKC phosphorylation sites, and S262-CKII phosphorylation sites (Zelenina, 2010). There is an additional PKC phosphorylation site in the human AQP1 amino acid sequence that CKI can phosphorylate. Additionally, Cys189 in AQP1 is a Hg^2+^ binding site, whereas Tyr186 is a TAE binding site, with the former limiting AQP1's water and ion permeability and the latter inhibiting its water permeability.

Several studies have demonstrated that PKA can phosphorylate AQP1 *in vitro* and that activators of PKA, such as cAMP and forskolin, can increase AQP1's water permeability (Smith et al., 2007). Vasopressin can enhance the water permeability of AQP1-transfected oocytes, and co-incubation and injection of 8-Bromo-cAMP into cells can also increase water permeability, which is hypothesized to occur *via* the cAMP pathway (Zelenina, 2010). Sakurada et al. (2004) demonstrated that 5% glucose enhanced AQP1 membrane transfer *via* the PKA pathway, which PKA-specific inhibitors could inhibit. However, several investigations have indicated that PKA does not promote AQP1 permeability *in vitro* (Wang et al., 2011). Sakurada et al. (2004) and Oshio et al. (2005) found that forskolin may increase CSF in wild-type mice and AQP1^−/−^ mice. It has not been established conclusively that PKA-regulated AQP1 phosphorylation occurs in the brain.

For PKC pathways, Zhang et al. (2007) showed that the water and ion permeability of AQP1 is regulated by PKC's short-term activity, involving two phosphorylation sites, T159 and T239. The most recent study discovered that, in addition to phosphorylation regulation, AQP1 could also be regulated by ubiquitin.

Chemical blockers of AQP1 that are permeable to water are constantly being developed, Hg^2+^ and TAE are constrained by their toxic effects, and recent studies have demonstrated that the Na+-K+-Cl-co-transport blocker and medullary loop diuretic Bumetanide has a slight inhibitory effect on the water permeability of AQP4. Furthermore, the body can tolerate a high amount (100 mg/kg). This finding paved the way for the development of further molecular compounds based on the structure of Bumetanide. According to Kim et al. (2010), the aryl-sulfonamide AqB013 significantly reduced the water permeability of AQP1 and AQP4, although its therapeutic potential remains unknown.

#### Gene regulation of AQP1

Changes in the external environment can affect the expression of the AQP1 gene (e.g., high seepage, low oxygen, etc.). AQP1 mRNA expression increased 2 fold in a high seepage environment (220 mM NaCl) but decreased in a low seepage environment (90 mM NaCl) (Bouley et al., 2009). Wang Feng et al. used primary culture of adult rabbit lumbar myelin nuclear cells, immunohistochemistry, and real-time quantitative PCR detection, and found significantly increased AQP1 expression in cells, which may be attributed to the increased expression of AQP1 to enhance oxygen delivery. 2-methoxyestradiol can inhibit hypoxia inducible factor-1α (HIF-1α) and vascular endothelial growth factor, hence influencing the expression of AQP-1.

This signal transduction pathway is critical for the regulation of AQP1 expression. In one study, Schwann cells inflated and AQP1 expression increased in response to hypoxia and HIF-1α may play a role in this process (Zhang et al., 2013), implying that the cGMP pathway regulates AQP1 expression. Ding et al. (2013) mimicked cardiopulmonary reflux using the guanosine cyclase inhibitor ODQ as a control. Compared to the control group, AQP1 mRNA expression fell by 39.7%, indicating that the cGMP pathway regulates AQP1 expression. McCoy and Sontheimer (2010) studied the expression of AQP1 following injury to active astrocytes using epidermal sting tests and discovered that the MEK1/2 pathway inhibitor U0126 might inhibit AQP1 expression. It was further demonstrated that cytokinin activates the protein kinase signaling pathway, inducing AQP expression. Experiments have also verified regulatory effects of JNK (Tie et al., 2012) and related pathways.

In both flanks of the *AQP1* gene, a substantial number of cis-regulatory elements have been identified. Although they do not encode any proteins themselves, they are critical for gene regulation. In conjunction with trans-acting factors, these cis-regulatory elements govern the transcription and translation of *AQP1* at the gene level. In the promoter region of *AQP1*, Moon et al. (1997) identified two glucocorticoid response elements. Steroid hormones can activate this response element and increase *AQP1* expression in murine leukemia cells.

Additionally, Stoenoiu et al. (2003) discovered that a high dose of corticosteroid (1.5 mg/kg) could stimulate AQP1 mRNA and protein expression in the peritoneum of rats. Simultaneously, the glucocorticoid receptor inhibitor Ru-486 worked on the glucocorticoid receptor and inhibited corticosteroid production. de Arteaga et al. (2011) discovered that high-dose corticosteroid therapy could increase the water level in patients undergoing peritoneal dialysis. These corresponding changes indicate that the glucocorticoid receptor and glucocorticoid response elements in the *AQP1* promoter region are intimately related to *AQP1* expression.

#### Translocation of AQP1

Owler et al. (2010) induced hydrocephalus in adult mice by injecting kaolin into the medullary cistern. In the experimental group, AQP1 was detected in the cytoplasm of CP epithelial cells, indicating a shift in the translocation of AQP1. Caveolin is a subunit of the membrane lipid cutting architecture that selectively transfers membrane proteins. It has been suggested that it is closely related to AQP1 translocation (Tie et al., 2012). Kobayashi et al. (2006) extended Western blotting findings to establish co-expression of AQP1 and caveolin in lipid cutting, and AQP1 localization indicated that AQP1 translocation might be associated with caveolin. Tietz et al. (2006) cultured bile duct epithelial cells *in vitro*, demonstrating that actin and microtubules may be involved in AQP1 translocation.

## AQP4 in hydrocephalus

It has been gradually understood that AQP4, a crucial component of CSF transport and interstitial fluid clearance, plays a role in iNPH in the impairment of CSF circulation, astrocyte hyperplasia, and impaired lymphatic circulation in the perivascular space and brain parenchyma. This leads to CSF accumulation, an inability to efficiently remove metabolic waste, and neurodegenerative changes. A promising prospective therapeutic target, AQP4 plays a variety of roles in iNPH. Contrary to other types of hydrocephalus, the expression of *AQP4* shows a decrease in iNPH, which may allow treatment routes that affect AQP4 activation to have a more noticeable effect in iNPH than in other types of hydrocephalus (Table 1).

**Table 1:** Main studies investigating AQP1 and AQP4 in humans.

**AQP**	**Author (years)**	**Research object**	**Result**
AQP1	Verkman (2008a)	AQP1 gene knockout animals	20% decrease in CSF fluid production and a 56% decrease in intracranial pressure
	Smith et al. (2007)	Communicating hydrocephalus	Lower expression of AQP1 on the apical membrane of the CPEpiC* of the ill choroid plexus
	Jeon et al. (2017)	Kaolin-injected hydrocephalus mice	AQP 1 protein expression reduced abruptly in the choroid plexus of rats with acute hydrocephalus and increased at later stages
	Wang et al. (2011)	Kaolin-injected hydrocephalus mice	A substantial number of AQP1 immune granules are present in the apical membrane of CPEpiC*
	Castañeyra-Ruiz et al. (2016)	iNPH patient	No significant increase in AQP1 levels in cerebrospinal fluid compared to the control group
AQP4	Ismail et al. (2009)	SAH mouse model	
	Papadopoulos and Verkman (2007)	AQP4 gene knockout mice	Increase in intracranial pressure and the moisture content of brain tissue
	Mao et al. (2006)	Kaolin-injected hydrocephalus mice	AQP-4mRNA expression increased significantly in the parietal lobe and hippocampus
	Bloch et al. (2006)	Congenital AQP4 protein deficiency rats	AQP4 protein can exacerbate hydrocephalus in kaolin-induced H-t gene deletion rats
	Tourdias et al. (2009)	Hemolytic lecithin-induced traffic hydrocephalus	AQP4 expression increased significantly in the foot process of astrocytes
	Shen et al. (2006)	Congenital hydrocephalus model in rats	Increased AQP4 expression in the blood-brain barrier, ependyma, and subpial astrocytes
	Eide and Hansson (2020)	iNPH	Decrease in AQP4 expression at the LM and EM levels of perivascular astrocyte terminals, which were inversely proportional
	Hasan-Olive et al. (2019b)	iNPH	Reduced density of AQP4 water channels in astrocytic endfoot membranes along cortical microvessels in patients with iNPH

AQP1, aquaporin 1; AQP4, aquaporin 4; iNPH, idiopathic normal pressure hydrocephalus; CPEpiC, choroid plexus epithelial cell.

### Regulation of AQP4 on CSF circulation

As another vital member of the water channel family, AQP4 is the most abundant aquaporin subtype in the central nervous system. It is primarily found in the protrusion membrane, glial boundary membrane, and ependyma of the foot processes of astrocytes surrounding capillaries, where it regulates the flow of water into and out of the brain *via* the CSF and blood. It exerts a considerable regulatory influence on CSF production and absorption (Hara-Chikuma and Verkman, 2008a,b; Verkman, 2008b). In cytotoxic cerebral edema caused by injuries such as water poisoning, cerebral infarction, or meningitis, AQP4-deficient mice had a better prognosis than wild-type mice (Verkman, 2008b). AQP4 deficiency bypasses the whole blood-brain barrier, hence lowering cytotoxic brain edema. In vascular cerebral edema mouse models such as cortical freezing, brain tumors, and intracranial rehydration fluid, AQP4-deficient animals demonstrated significantly more brain swelling than wild-type mice, most likely because AQP4 in wild mice expedited the outflow of water from edema tissue. Additionally, in hydrocephalus, AQP4 regulates CSF absorption (Kusayama et al., 2011). Matthew et al. established the first mouse model of subarachnoid hemorrhage by injecting autologous blood into the basal cistern, simulating how high-pressure blood enters the CSF in human subarachnoid hemorrhage. The results indicated that AQP negative mice had more evident brain swelling and a poorer clinical prognosis than controls, primarily because AQP4 encouraged the evacuation of excess water in brain tissue *via* the compromised blood–brain barrier (Ismail et al., 2009).

Additional evidence linking AQP4 protein to CSF production and absorption has been revealed. Papadopoulos and Verkman (2007) observed an increase in intracranial pressure and the moisture content of brain tissue in gene knockout mice. When *AQP4* knockout mice were compared to wild-type mice, they had significantly reduced water penetration and a larger ventricular volume, implying that AQP4 is required for cross-substantive absorption of CSF. AQP4 demonstrates its impact on the occurrence and progression of hydrocephalus in a number of animal models of the condition. Mao et al. (2006) discovered that AQP4 mRNA expression increased significantly in the parietal lobe and hippocampus when kaolin-induced hydrocephalus was studied in mice. This could be because peripheral AQP4 expression is more easily activated in the brain's parietal lobe, and the parietal lobe and hippocampal regions have a lower water content than other regions. Bloch et al. (2006) also discovered that AQP4 can exacerbate hydrocephalus in kaolin-induced *H-t* gene deletion rats (congenital AQP4 protein deficiency rats) by decreasing the rate of extracellular fluid clearance across the blood-brain barrier, indicating that AQP4 plays a beneficial role in increasing the blood-brain barrier clearance rate and preventing the accumulation of excess water in the ventricles. Tourdias et al. (2009) calculated the apparent diffusion coefficient in rats with hemolytic lecithin-induced traffic hydrocephalus. After 2 weeks, it was discovered that AQP4 expression increased significantly in the foot processes of astrocytes and that there was a significant correlation between AQP4 levels, apparent diffusion coefficient, and ventricular volume. Shen et al. (2006) used the congenital hydrocephalus model in rats and discovered that 8-week-old rats had increased AQP4 expression in the blood–brain barrier, ependyma, and subpial astrocytes. Additionally, it was hypothesized that increased AQP4 expression might be a compensatory response to CSF absorption in static hydrocephalus. In normal circumstances, CSF circulation is hampered after hydrocephalus. AQP4 expression rises as a result of compensatory trans-parenchymal absorption of CSF, and accumulating CSF is absorbed into the circulation *via* an AQP4-dependent transcellular pathway.

### AQP4 in iNPH

In iNPH, the situation is substantially different, possibly due to a failure of the compensating process or a weakness in specific CSF linkages. Although it is commonly believed that AQP4 levels are elevated in patients with hydrocephalus, it was found that they decreased in iNPH (Eide and Hansson, 2018, 2020; Arighi et al., 2019; Hasan-Olive et al., 2019a,b). Eide and Hansson (2020) found a significant increase in the percentage of fibrin (fibrinogen) areas in iNPH cerebral cortex exosmosis. At the same time, AQP4 expression was decreased in the vicinity of blood vessels. Immunohistochemistry revealed a decrease in AQP4 expression at the light and electron microscopy levels of perivascular astrocyte terminals, which were inversely proportional.

In the research of Hasan-Olive et al. (2019b), AQP4 immunogold cytochemistry study of cortical brain samples from 30 patients with iNPH and 12 control participants revealed lower AQP4 density in the perivascular astroglial end-feet of patients. In brains from individuals with aneurysms, epilepsy, and cancer, astroglial AQP4 density facing the neuropil remained unaltered, as did perivascular AQP4 density. This link could point to a role for inflammation-induced AQP4 depolarization in iNPH pathogenesis, in which reduced perivascular AQP4 expression limits glymphatic fluid flux, increasing beta amyloid buildup and ventriculomegaly. In iNPH, the expression of AQP4 is decreased in the vicinity of blood vessels, resulting in issues with lymphatic circulation, waste disposal, and neurodegeneration (Hasan-Olive et al., 2019b).

There is no apparent explanation for why AQP4 expression differs so much between common hydrocephalus and iNPH. Recent research suggests that this phenomenon may be linked to inflammation. It was related to lymphatic malfunction in the early stages of inflammation by Reeves et al. (2020). In fact, investigations in both humans (Sosvorova et al., 2015) and animals (Sosvorova et al., 2015; Chaudhry et al., 2017) have identified a connection between perivascular space inflammation and the development of iNPH. In iNPH, the neuroinflammatory response is highly noticeable (Lolansen et al., 2021), and the level of inflammatory cytokines indicated by TNF in the CSF fluid is dramatically altered (Wang et al., 2020). However, we can demonstrate that AQP4 plays a role in CSF transport and interstitial fluid clearance, and that its reduction in iNPH has an effect on CSF circulation and lymphatic metabolic abnormalities.

### Effect of AQP4 on the hyperplasia of astrocytes

The AQP4 protein is favorably linked with astrocyte development. Tomas-Camardiel et al. (2005) described an intracerebroventricular injection of lipopolysaccharide as a model of hemorrhagic brain edema. It was revealed that activated astrocytes expressed a high quantity of AQP4. Saadoun et al. (2009) discovered that pricking the cortex decreased astrocyte growth in AQP4 rats with congenital gene deficits, indicating that AQP4 promoted astrocyte proliferation. Zhou et al. (2008) also discovered that the absence of AQP4 caused a significant decrease in the expression of GFAP in the surrounding brain microvessels when examining rats with congenital AQP4 deficits, and GFAP was positively linked with the degree of astrocyte proliferation.

GFAP interacts closely with membrane anchoring proteins, allowing GFAP fibers to pull the membrane back when they retract in response to changes in signaling molecules. That is, as GFAP fibers retract, squeezing water out of the cytoplasm of the processes, the interactions between the anchor proteins and GFAP fibers cause volemic reduction (Wang and Hatton, 2009). The expression of volume-regulating proteins and their interactions with membrane anchoring proteins determine the volemic influence on GFAP's guiding role. AQP4 expression is synchronized with the levels and spatial distribution of GFAP, as expected for a volume-regulating protein (Wang and Hatton, 2009; Li et al., 2020). Furthermore, Wang X. et al. (2021) found TGN-020's ability to prevent GFAP retraction surrounding vasopressin neurons during hyposmotic challenge (20 min) and hyperosmotic stress suggesting that AQP4 plays a role in GFAP retraction. AQP4 is functionally connected with several ion channels and transporters, and its opening for water flux is accompanied by changes in ion concentration in astrocytes, which might affect GFAP metabolism and assembly, as previously discussed. By coupling with transient receptor potential vanilloid 4, Benfenati et al. (2011) and Stokum et al. (2018) discovered that AQP4 can increase intracellular Ca^2+^; however this coupling is not required (Mola et al., 2016). Increased intracellular Ca^2+^ levels are required for GFAP depolymerization *via* protein kinase A and other signaling pathways (Petković et al., 2017), suggesting that AQP4 might reduce GFAP filament extension.

However, astrocyte growth also contributes to alterations in brain tissue function after hydrocephalus. When hydrocephalus occurs, the ventricle system extends the ventricular wall due to the accumulation of CSF. Astrocyte proliferation also occurs surrounding the ventricle, creating compensatory enlargement of the ventricle, causing CSF extravasation into the brain tissue around the ventricle, causing brain white matter edema and white matter tissue deterioration. When the CSF content is further increased, the elasticity of the brain tissue reduces due to compression, the cerebral cortex gets thinner, leading to brain atrophy, and these pathological changes result in neurological dysfunction, indicating that AQP4 stimulates GFAP and astrocyte proliferation, which may be the source of the changes in neurological function following hydrocephalus.

In patients with iNPH, Eide and Hansson (2018, 2020) detected nerve cell degeneration, astrocyte proliferation, and decreased expression of AQP4 and Dp71. In comparison to reference patients, cortical biopsies of individuals with iNPH revealed increased astrocyte proliferation and lower expression of AQP4 and Dp71 in astrocyte perivascular terminals in the foot and certain surrounding neural canals. The degree of astrocyte proliferation was substantially linked with a decrease in AQP4 and Dp71 levels in the astrocytes' perivascular terminal feet. Additionally, all patients with iNPH have bulging nerve cell bodies and neurodegeneration. While astrocytosis is associated with activated macrophages and microglia, the inflammatory marker CD68 did not differ between iNPH and REF. Dp71 is the major dystrophin subtype in the brain, connecting the cytoskeleton, cell membrane, and extracellular matrix dystrophin-related protein complex (DAPC) to anchor AQP4 and ion channels, with a preference for the lipid raft surrounding the astrocytes' terminal foot. It is required for adequate fluid transport between the cerebrovascular system, the interstitial fluid space, and astrocytes. AQP4 appears to be polarized toward the terminal foot of perivascular astrocytes in response to the components of the DAPC (Nicchia et al., 2008; Enger et al., 2012; Camassa et al., 2015). As Eide and Hansson (2018) previously established, dystrophin deficiency causes instability in the interface between the cytoskeleton and the mass membrane, impairing signal transduction. Experiments causing the loss or displacement of AQP4 or DAPC can result in aberrant brain water flux and disruption of intermediate metabolism, which can eventually result in the slow development of neurodegeneration (Nagelhus and Ottersen, 2013; Pavlin et al., 2017). Patients with iNPH who reacted to CSF fluid shunts had aberrant intracranial throbbing pressure, indicating impaired compliance. The primary histopathological findings were the proliferation of astrocytes and the subsequent drop of AQP4 and Dp71 at the astrocytes' perivascular terminal foot. According to Eide, alterations in the AQP4 and Dp71 complexes are related to sub-ischemia, often in tissue (Eide and Hansson, 2018).

**Figure 2:**
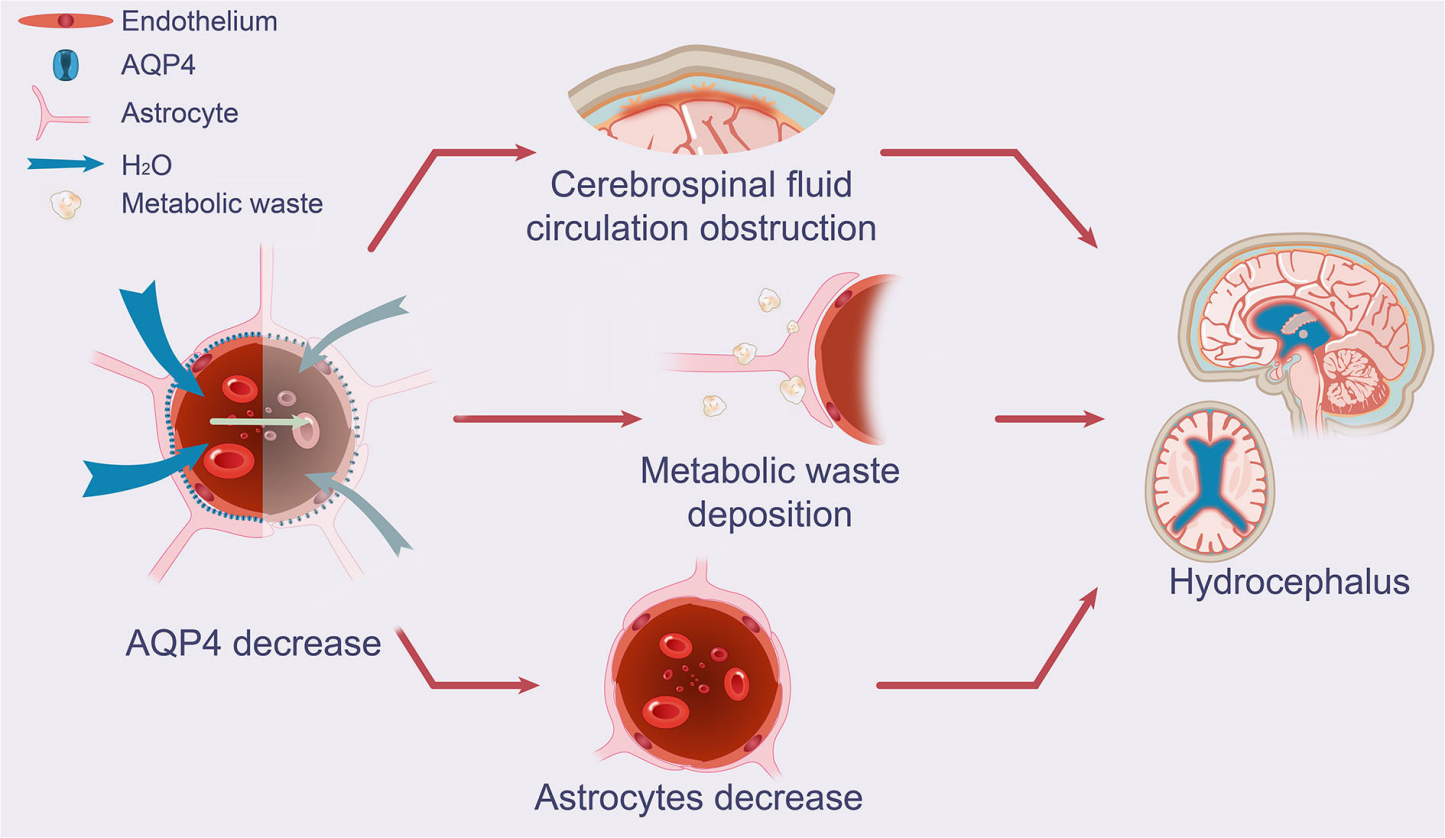
Effect of AQP4 on potential glymphatic dysfunction and growth of astrocytes in iNPH. The schematic drawing demonstrates decreases in AQP4 and its function in iNPH, which leads to diminished CSF periarterial inflow, CSF-ISF exchange, and perivenous outflow of CSF mediated by AQP4 depolarization, as seen in patients with iNPH. Increased concentrations of neuronal metabolic waste products in the brain interstitial space result from the decreased exchange of CSF and ISF. A decrease in AQP4 in the end-feet of astrocytes near arteries was substantially associated with astrocyte growth and CSF circulation obstruction. AQP4, aquaporin 4; iNPH, idiopathic normal pressure hydrocephalus; CSF, cerebrospinal fluid; ISF, interstitial fluid.

Astrocytosis may contribute to the decreased intracranial compliance (pressure-volume reserve capacity) observed in patients with iNPH, as indicated by higher pulse wave intracranial pressure (Eide and Sorteberg, 2010, 2016; Eidsvaag et al., 2017). Mechanical resistance and hardness are known to be significantly increased by iNPH (Lu et al., 2011). Glia, including astrocytes, account for more than half of the total number of cells and brain volume in adults (Zhan et al., 2020). Fibrin (fibrinogen) has been shown to excite astrocytes and small astrocytes, implying that they may contribute to the progression of neurodegenerative changes and cognitive deficiencies (Figure 2) (Cortes-Canteli et al., 2010; Schachtrup et al., 2010; Merlini et al., 2019).

### Effect of AQP4 on lymphatic system drainage disorder

In contrast to other types of hydrocephalus, the decrease in AQP4 in iNPH and Alzheimer's disease is related to the progressive loss of perivascular units responsible for water exchange (Reeves et al., 2020). This finding may indicate a different sort of hydrocephalus, which would account for the normal intracranial pressure and low AQP4 levels. Indeed, there may be no loss of brain parenchyma in traffic hypertension hydrocephalus but rather an accumulation of CSF due to constriction of the perivascular space (Blasco et al., 2014; Schmidt et al., 2016). As a result, as intracranial pressure increases, AQP4 levels may rise, first in the blood, then in the CSF (Castañeyra-Ruiz et al., 2013; Blasco et al., 2014). On the other hand, iNPH does not result in an increase in intracranial pressure despite the increasing buildup of amyloid beta, loss of brain parenchyma, and progressive lymphatic system dysfunction. Brain atrophy could be the cause. Hence, patients with iNPH may have low AQP4 concentrations in their CSF due to a lack of AQP4 expression and the loss of functional vascular units (Eide and Hansson, 2018, 2020; Arighi et al., 2019; Hasan-Olive et al., 2019a,b).

In rodents, AQP4 deficiency results in water build-up in the brain parenchyma (Haj-Yasein et al., 2011; Vindedal et al., 2016), decreased perivascular cerebrospinal and interstitial fluid circulation, and poor amyloid beta clearance (Iliff et al., 2012). These findings are equally perplexing as those regarding the blood–brain barrier, as AQP4 defective mice demonstrate normal blood–brain barrier integrity (Saadoun et al., 2009; Eilert-Olsen et al., 2012). As a result, a decrease in AQP4 surrounding blood vessels may directly affect the flow of fluid around blood vessels and the brain's steady state.

According to MRI studies, individuals with iNPH showed decreased lymphatic function, higher accumulation, and a reduced clearance rate for the intrauterine injectable contrast agent gadobutrol (Ringstad et al., 2017; Eide and Ringstad, 2019). MRI revealed reduced vascular side gaps and lymphatic circulation in patients with iNPH (Eide and Ringstad, 2019). Gadobutrol circulates through the brain's large soft meningeal arteries. In individuals with iNPH, cerebral blood flow perfusion and brain metabolic levels were decreased (Eide and Stanisic, 2010; Calcagni et al., 2013; Ziegelitz et al., 2016).

Hasan-Olive et al. (2019b) confirmed for the first time that AQP4 expression in patients with iNPH dropped dramatically in astrocytic end-foot membranes facing capillary endothelial cells than in end-foot membranes facing neuropil. Reduced AQP4 around blood vessels may impair fluid and lymphatic circulation in the brains of patients with iNPH. Given the strategic location of the AQP4 water channel between the blood and the interstitial fluid, it is fair to assume that a CSF shunt will improve liquid flow through the AQP4 water channel. This increases the availability of nutrients and oxygen, as well as waste elimination, in the brains of patients with iNPH (Tan et al., 2021).

### The expression and regulation mechanism of AQP4

Numerous studies have been conducted to elucidate the numerous endogenous mechanisms of AQP4 control, as well as the potential for pharmacological modulation. Four distinct regulation mechanisms have been extensively studied: microRNA (miRNA)-mediated regulation of gene expression, phosphorylation-mediated regulation of AQP4 channel gating/transport, heavy metal-ion-mediated regulation of water permeability, and small molecular inhibitor-mediated regulation of water permeability.

MiRNA targeting of AQP4 has become an area of research and development in AQP4 regulation (Gomes et al., 2018). The miRNA is an endogenous single-stranded RNA sequence obtained from most of its inclusions that function primarily in the degradation or inhibition of mRNA translation (Eide and Sorteberg, 2010, 2016). Recently, numerous studies have established a relationship between specific miRNAs and therapeutic AQP4s (miR-224/miR-19a, miR-29b, miRNA-145, miRNA-320a, miRNA-130a, and miRNA-130b).

Phosphorylation is an effective post-translational modification method. It can modulate protein function or expression by influencing protein structure, protein-protein interaction, and protein transport labels (Nesverova and Tornroth-Horsefield, 2019). Phosphorylation of AQP4 is required for its transport and subcellular localization and may be involved in channel gating (Kadohira et al., 2008; Nesverova and Tornroth-Horsefield, 2019). While the majority of recent research has been on S111, S180, and S276, there are still more putative phosphorylation sites, including S285, S315, S316, S321, and S322, whose function is unknown (Lundby et al., 2012; Nesverova and Tornroth-Horsefield, 2019). However, tests in oocytes utilizing mutant rAQP4 protein showed that phosphorylation of the COOH terminal serine residues S315, S316, S321, and S322 did not affect transport or channel gating (Assentoft et al., 2014). The S315 phosphorylation location discovered in rats is not conserved in human AQP4 (Lundby et al., 2012) as serine is converted to glutamine. The effect of phosphorylation on AQP4 remains an intriguing area of research, with numerous processes and routes yet unknown.

The Kidins220–SNX27–retromer–AQP4 pathway was discovered as a regulatory mechanism for brain AQP4 expression and its participation in brain ventricular enlargement and hydrocephalus by Puerto et al. We detected an unexpected relationship between SNX27-retromer downregulation and AQP4 lysosomal degradation caused by the loss of Kidins220, as well as a dramatic drop in Kidins220 and AQP4 expression at the ependymal barrier in patients with iNPH (Del Puerto et al., 2021).

Mercury is at least one of the metals that regulates the majority of AQPs (Ximenes-da-Silva, 2016; Abir-Awan et al., 2019). It inhibits practically all AQPs except AQP6 and acts predominantly on AQPs (Yasui et al., 1999). Experiments with protein liposomes demonstrated that the Hg^2+^ binding site on AQP4 is located within the cell, which means that Hg^2+^ cannot reach its direct binding site on AQP4 in oocytes or most cell models and, thus, cannot regulate its activity (Yukutake et al., 2008; Yukutake and Yasui, 2010). On the other hand, the mercury ion can covalently attach to the C178 residue of the D ring of rAQP4's intracellular domain, exposing the Hg^2+^ binding site to the external solution and perhaps causing AQP4's conformational change (Yukutake et al., 2008; Ximenes-da-Silva, 2016). *In vivo*, however, the effect was not inhibition but a significant increase in AQP4 protein production, most seen in reactive astrocytes.

Additionally, zinc and copper were found to be inhibitors of rAQP4 in protein liposomes (Yukutake et al., 2009). Not all metal ions that affect AQP4 activity operate directly; some act indirectly by initiating phosphorylation pathways, leading to AQP4 expression or water permeability changes. These characteristics are shared by other materials such as lead (Gunnarson et al., 2005), manganese (Rao et al., 2010), and copper (Qing et al., 2009).

A small-molecule inhibitor is a compound that inhibits AQP4 without the need for metal ions. It is critical to identify small molecule AQP4 antagonists as AQP4 activity or expression is associated with various illnesses, and small molecule inhibitors can inhibit AQP4 without generating metal ion toxicity. Experimental verification tests have been performed on acetazolamide, validic acid, topiramate, ethoxzolamide, zonisamide, phenytoin, lamotrigine, and sumatriptan, among other drugs.

The methodological diversity is one of the most perplexing features of AQP4 regulation. Although *in vitro* techniques vary considerably, various miRNAs, heavy metal ions, and two small molecule inhibitors, acetazolamide and TGN-020, all demonstrate promise in regulating AQP4.

## Conclusions

While the pathogenesis of iNPH remains a complicated subject, AQPs appear to be a feasible avenue for treatment. As AQP4 and AQP1, which have been studied for over a decade, are highly expressed in brain tissue and participate in the pathophysiological processes underlying related brain diseases, their critical role in maintaining the balance of brain tissue water in the nervous system and CSF circulation is undisputed. Numerous experimental studies have corroborated the pattern of increased AQP1 and decreased AQP4 protein expression in iNPH (Table 2).

**Table 2:** AQP1 and AQP4 in iNPH.

	**AQP1**	**AQP4**
Positional distribution in the nervous system	The top and outer parts of the vein epithelial cells	Plasma membrane, glial boundary membrane and ependyma of foot process of astrocytes around capillaries
Effects on cerebrospinal fluid circulation	The positional distribution of the top AQP1 facilitates cross-cell transport of water molecules during the development of cerebrospinal fluid	Accumulated cerebrospinal fluid be absorbed into the bloodstream through AQP4-dependent transcellular pathway
In the genetic knockout animal model	Cerebrospinal fluid production is reduced and intracranial pressure is reduced	The cerebrospinal fluid increases, intracranial pressure increases, and the permeability of water in brain tissue is greatly reduced
In iNPH patients	Abnormally elevated	Abnormally decreased
Mechanisms that act in brain tissue in iNPH patients	Mostly like other types of hydrocephalus, the polarity distribution of AQP1 is protected from extinction	Mostly unlike other types of hydrocephalus patients, may be blocked lymphatic circulation and hyperplication of glial cells
Expression and adjustment	Multi-level adjustment	MicroRNAs, phosphorylation, heavy metal ions, small molecule inhibitors

AQP1, aquaporin 1; AQP4, aquaporin 4.

AQP1, through its polar distribution, mainly plays a role in promoting CSF production, maintaining the balance of hydrocephalus, and participating in pathological processes. When CSF levels are elevated in iNPH, defective internal protective mechanisms permit AQP1 to continue to play a role in boosting generation, suggesting that we can govern linked brain illnesses by regulating AQP1. In the three levels of AQP1 regulation, the most effective control is the translation level of transcription adjustment and AQP1 itself. Transposing during the pathologic process complicates its management. Thus, in the future, we will require a more accurate understanding and in-depth investigations of the regulation mechanisms of AQP1 expression to develop a more effective method for AQP1 regulation. At the same time, the mechanisms causing changes in its polar distribution requires further investigation; the causes of abnormalities in AQP1-related protection mechanisms—the mechanism of iNPH occurrence—from a more comprehensive perspective are necessary for a deeper level understanding essential for the diagnosis and treatment.

AQP4 contributes to the development of many neurological illnesses through various biological effects, including an increase in the pace of blood-brain barrier clearance and a decrease in excess water storage in ventricles. AQP4 expression is increased in other types of hydrocephaly to drain extra CSF. However, its expression is frequently reduced in patients with iNPH, resulting in poor lymphatic circulation, waste clearance, and consequent neurodegeneration. As a result, AQP4 may be a critical therapeutic target in the treatment of neurological disorders, and with the advancement of research, the study of AQP4 gene polymorphisms and a range of neurological diseases is also advancing.

The role of AQPs in iNPH has gradually come to the attention of more researchers, with the majority of research focusing on AQP4 and AQP1, and the mechanisms of damage in iNPH have been gradually revealed, although there is still a need for further exploration. Applications in the diagnosis and treatment of iNPH have also received an increasing amount of interest. However, as evidenced by recent studies, it appears to be impossible to detect AQP1 and AQP4 in CSF to serve as diagnostic markers. As far as enzyme-linked immunodetection assays are concerned, it appears that AQP1 and AQP4 will not enter CSF. Additional opportunities, such as targeted imaging enhancement, are also worth investigating. Following acetazolamide, the AQP4 activator EPO has emerged as a potential therapy option and has so far shown promising results in animal models. Despite the fact that AQP1 inhibitors have been cited in a few publications, there has been no advancement in their use at this time. In subsequent stages of this research, there are numerous points to consider as well. The distribution of AQP1 and AQP4 in the brain is not confined to a single location. While AQP4 was discovered in the CP, AQP1 was also discovered in capillary astrocytes. The issue remains of how to direct medications to the precise locations where they will be most beneficial. The usefulness of using AQPs in targeted treatments for neurological illnesses has been made more evident.

## Author contributions

ZZ and JH collected the related manuscript. ZT, JH, YC, YW, CW, CT, and JL drafted and revised the manuscript. GX participated in the design of the review, helped to draft, and revised the manuscript. All authors read and approved the final manuscript.

## Funding

This work was supported by National Natural Science Foundation of China (No. 82171347), Hunan Provincial Natural Science Foundation of China (No. 2019JJ50949), the Scientific Research Project of Hunan Provincial Health Commission of China (No. 202204040024), and the Students Innovations in Central South University of China (No. S2022105330687).

## Conflict of interest

The authors declare that the research was conducted in the absence of any commercial or financial relationships that could be construed as a potential conflict of interest.

## Publisher's note

All claims expressed in this article are solely those of the authors and do not necessarily represent those of their affiliated organizations, or those of the publisher, the editors and the reviewers. Any product that may be evaluated in this article, or claim that may be made by its manufacturer, is not guaranteed or endorsed by the publisher.
